# Prognostic Role of the Endothelial Activation and Stress Index (EASIX) in Functional Outcomes and Mortality After Acute Ischemic Stroke: A Retrospective Pilot Cohort Study

**DOI:** 10.3390/jcdd13020066

**Published:** 2026-01-27

**Authors:** Michail Makris, Eleftheria Ztriva, Eleni Gavriilaki, Vasileios Patriarcheas, Vasiliki Gougoula, Michail Giannakakis, Alexandros Tselepis, Georgios Ntaios, Christos Savopoulos, Georgia Kaiafa

**Affiliations:** 1 First Propaedeutic Department of Internal Medicine, AHEPA University Hospital, Aristotle University of Thessaloniki, Stilponos Kyriakides 1 Str., 54636 Thessaloniki, Greece; michaelmakris4@gmail.com (M.M.); elztriva@gmail.com (E.Z.); vpatriar@gmail.com (V.P.); vasilikigougoula@gmail.com (V.G.); mixalisgiann@hotmail.com (M.G.); gntaios@auth.gr (G.N.); chrisavopoulos@gmail.com (C.S.); 2 Second Propedeutic Department of Internal Medicine, Hippokration General Hospital, Aristotle University of Thessaloniki, Konstantinoupoleos 49 St., 54942 Thessaloniki, Greece; elenicelli@yahoo.gr; 3 Atherothrombosis Research Centre/Laboratory of Biochemistry, Department of Chemistry, University of Ioannina, 45110 Ioannina, Greece; atselep@uoi.gr

**Keywords:** ischemic stroke, endothelial dysfunction, EASIX, small vessel disease

## Abstract

Background: Endothelial dysfunction is a key player in stroke pathophysiology. The Endothelial Activation and Stress Index (EASIX) is a biomarker of endothelial injury validated in hematology, sepsis, and cardiovascular cohorts; however, its prognostic role in stroke remains unclear. This retrospective cohort study aims to provide preliminary evidence on the potential utility of EASIX levels as a biomarker for assessing stroke severity and predicting outcomes. Methods: We retrospectively studied 100 patients aged ≥ 18 years admitted with acute ischemic stroke (AIS) or transient ischemic attack (TIA) between January 2020 and July 2024. EASIX was calculated on admission as LDH × creatinine/platelets. Outcomes included in-hospital and 12-month mortality, stroke severity assessed by the NIHSS score, and disability assessed as a modified Rankin score (mRS). Results: Median age was 82 years; 56% were female. The in-hospital and 12-month mortality rates were 47.9% in patients with AIS and 17.2% in patients with TIA, respectively. Overall, EASIX was not associated with NIHSS, mRS, or mortality in the total cohort. Ιn the subgroup of patients with small vessel disease (*n* = 10), higher EASIX was associated with worse mRS at 12 months (β = 2.383, *p* = 0.02) and increased mortality (β = 0.653, *p* = 0.02). EASIX correlated positively with WBC (*p* < 0.001) and CRP (*p* = 0.01). Female sex was associated with lower EASIX values. Conclusions: EASIX was not associated with outcomes in the overall AIS/TIA cohort, but it demonstrated potential prognostic relevance in small vessel disease (SVD), which has not been reported previously in the literature. Further prospective research is warranted to validate the potential association between systemic endothelial stress and small vessel disease before the implementation of EASIX as a prognostic tool in patients with stroke due to SVD.

## 1. Introduction

The Global Burden of Disease (GBD) estimates that nearly 12 million new stroke cases occur annually, with over 100 million individuals having experienced a stroke in their lifetime globally. Ischemic stroke is the most common type of stroke, accounting for approximately 81–87% of all strokes as a result of thrombotic or embolic occlusion of cerebral arteries [1,2]. The pathophysiology of ischaemic stroke is intricate and encompasses a series of events that extend beyond the initial artery occlusion, which serves as a trigger. This process includes the activation of inflammatory pathways, generation of oxidative stress, enhancement of platelet aggregation, and disruption of the blood–brain barrier. The processes involved contribute to thrombus formation and the exacerbation of brain injury through mechanisms including excitotoxicity, immune cell infiltration, and microvascular failure [3]. Endothelial dysfunction has an important role in this cascade of events, as the endothelium plays a crucial role in maintaining vascular tone, barrier integrity, and hemostasis, and when compromised, it leads to pro-thrombotic states, leukocyte recruitment, and the formation of cerebral edema [3,4].

Endothelial dysfunction has emerged as a central pathophysiological mechanism across a wide spectrum of cardiovascular, inflammatory, and infectious diseases, reflecting its pivotal role in maintaining vascular homeostasis and regulating systemic inflammation [5,6]. The prognosis of ischemic stroke is profoundly influenced by the extent of neuroinflammation and the integrity of the blood–brain barrier (BBB), both of which are critically regulated by endothelial activity. Disruption of the BBB triggers infiltration of inflammatory cells, oxidative stress, and secondary neuronal injury—processes tightly linked to endothelial activation and dysfunction. Experimental studies have shown that endothelial-derived molecules modulate leukocyte adhesion, permeability, and neuroinflammatory signaling, thereby shaping the severity and outcome of cerebral ischemia [7,8]. This pathophysiological interdependence underscores the importance of identifying reliable circulating biomarkers that reflect endothelial activation and vascular inflammation. The systematic identification and validation of such biomarkers not only deepens our understanding of disease pathophysiology but also advances the development of precision medicine by enabling the stratification of patients based on individualized endothelial and inflammatory profiles, thereby informing and optimizing personalized therapeutic strategies [9,10]. Within this framework, the Endothelial Activation and Stress Index (EASIX) represents a readily available composite marker that integrates endothelial and inflammatory stress responses, offering potential utility for individualized prognostic assessment in acute ischemic stroke.

The Endothelial Activation and Stress Index (EASIX) is a biomarker based on the calculation of three simple laboratory parameters: Lactate dehydrogenase (LDH) multiplied by creatinine divided by platelet count [11,12,13]. EASIX has been demonstrated in various clinical scenarios as an indicator of endothelial dysfunction, including acute graft-versus-host disease, Diffuse Large B-Cell Lymphoma (DLBCL), in CAR-T therapy recipients, COVID-19, Coronary Artery Disease (CAD), and in ICU-admitted Acute Myocardial Infarction (AMI) patients [14,15,16,17,18,19]. With regard to the role of EASIX in patients with stroke, EASIX was associated with stroke prevalence and all-cause mortality in the overall stroke population, but there is no data on whether this association differs between subtypes of ischemic stroke and whether the EASIX is associated with stroke severity and disability [20].

The aim of the present study is to examine the correlation between EASIX levels and stroke severity, in-hospital and 12-month all-cause mortality, and 12-month disability in the overall stroke population and in subtypes of ischemic stroke.

## 2. Materials and Methods

This retrospective study enrolled patients with age ≥ 18 years who were diagnosed with AIS or TIA in the AHEPA tertiary University Hospital in Thessaloniki, Greece, between January 2020 and July 2024. All study participants provided informed consent, the study was performed according to the Declaration of Helsinki principles, and the ethical review board of Aristotle University of Thessaloniki revised and approved the study protocol (No. 09/2022).

Exclusion criteria were intracranial hemorrhage, malignancy, hematologic disorder, acute central nervous system or systemic infection, and severe renal (eGFR < 30 mL/min) or hepatic disease. Furthermore, patients who underwent intravenous thrombolysis or mechanical thrombectomy were excluded from the study.

Stroke etiology was classified according to the TOAST criteria, based on standardized clinical assessment, neuroimaging, vascular imaging, and cardiac evaluation, with each patient assigned to a single etiological category corresponding to the most likely stroke mechanism.

**Figure 1:**
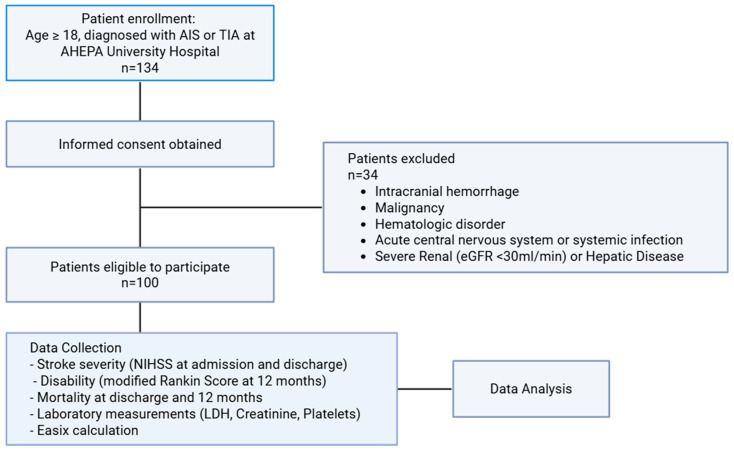
Flowchart.

The outcomes assessed included stroke severity, assessed with the National Institutes of Health Stroke Scale (NIHSS) score at admission (NIHSSadm) and at discharge (NIHSSdis) [21,22,23]; disability, assessed as a modified Rankin Score at 12 months; and all-cause mortality at discharge and at 12 months. Figure 1 shows the flowchart of the study.

EASIX was calculated using the formula (lactate dehydrogenase [LDH] [units per liter] × creatinine [milligrams per deciliter]/platelets [PLTs] [× 10^9^ per liter]). Both LDH and creatinine were measured in the DxC 700 AU chemistry analyzer (Beckman Coulter, Brea, CA, USA), using the kinetic UV method and a colorimetric enzymatic method, respectively. Platelets levels were measured in the XN-10, Sysmex Hematology analyzer (Sysmex Corporation, Kobe, Hyogo, Japan), using fluorescent flow cytometry.

All included patients received standard-of-care medical management for AIS or TIA, in accordance with current international guidelines.

### Statistical Analysis

Statistical analysis of the data was performed with SPSS 28.0 (IBM SPSS Statistics for Windows, Version 28.0.; IBM Corp., Armonk, NY, USA). For the presentation of variables with a normal distribution, means with standard deviations (SDs) were used, while for those with non-normal distribution, medians and IQR intervals (Q1–Q3) were used. Univariable regression was used to demonstrate correlation of EASIX to baseline characteristics and outcomes. We did further subgroup analysis between AIS and TIA, as well as among stroke subtypes according to the Trial of Org 10172 in Acute Stroke Treatment (TOAST) criteria [24].

## 3. Results

### 3.1. Baseline Characteristics

The study included 100 patients with AIS (*n* = 71) or TIA (*n* = 29), with a median age of 82 years, of whom 56 were females. Among the 71 patients with ischemic stroke, 33 (46.48%) had large artery atherosclerotic, 27 (38.03%) cardioembolic, 10 (14.09%) lacunar, and 1 (1.41%) undetermined. No statistically significant differences were observed in age or comorbidities between AIS and TIA groups. The median duration of hospitalization for AIS was 7 days, and for TIA it was 4. The median NIHSS at admission for patients with AIS was 8. Table 1 summarizes the baseline characteristics of the recruited patients.

### 3.2. Correlation of EASIX with Baseline Characteristics

**Figure 2:**
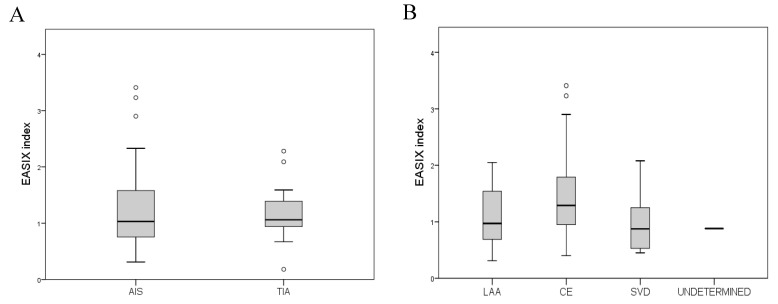
(**A**) Boxplot of EASIX Distributions in AIS vs. TIA Patients. (**B**) Boxplot of EASIX values across different ischemic stroke subtypes: large artery atherosclerosis (LAA), cardioembolic (CE), small vessel disease (SVD), and undetermined etiology. Median values, interquartile ranges, and outliers are displayed.

In the overall cohort, at admission, the median EASIX was 1.06. There was no difference in EASIX between patients with AIS and TIA, nor among different stroke types (Figure 2A). Also, there was no difference between SVD and non-SVD strokes, nor between cardioembolic and non-cardioembolic strokes (Figure 2B). Table 2 demonstrates correlations of EASIX with baseline characteristics with univariable linear regression models.

**Figure 3:**
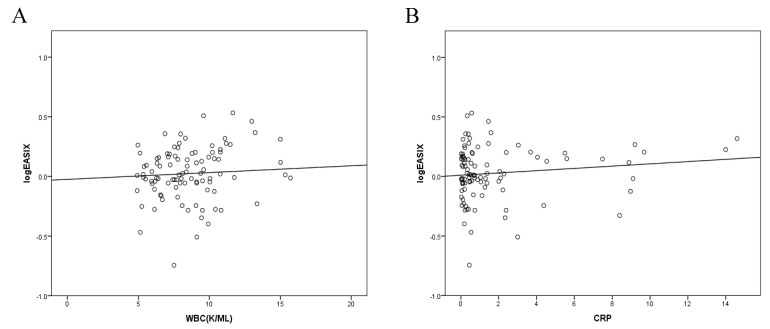
(**A**) Scatter plot illustrating the relationship between white blood cell count (WBC, K/µL) and log-transformed EASIX (logEASIX). A modest positive linear association is observed, as indicated by the regression line. (**B**) Scatter plot depicting the correlation between C-reactive protein (CRP) levels and log-transformed EASIX (logEASIX). A weak positive linear association is observed, as shown by the regression line.

In AIS, female sex demonstrated a significant negative association with EASIX (B = –0.115, *p* = 0.03). Higher WBC and CRP levels were also correlated to higher EASIX. (Figure 3). The recorded vascular risk factors like Arterial Hypertension, Diabetes Mellitus, Dyslipidemia, or smoking, as well as the rest of the laboratory results, showed no significant correlation to EASIX.

### 3.3. Correlation of EASIX with Outcomes

The median mRS at 12-month follow-up for AIS was 5, and for TIA 2, respectively. There were 34 deaths (47.9%) for AIS and for TIA 5 (17.2%) at 12-month follow-up. In the overall cohort of AIS and TIA, higher EASIX was not significantly associated with any of the outcomes (Table 3). In the subgroup analysis, we identified significant associations in patients with SVD and 12-month mortality (B = 0.653, *p* = 0.02). Higher EASIX was significantly correlated with mRS at 12 months (B = 2.383, *p* = 0.02) and with 12-month mortality (B = 0.653, *p* = 0.02). No such correlations were observed in the non-SVD group (Table 4).

## 4. Discussion

In this retrospective study, we investigated the prognostic role of EASIX in patients who presented with Acute Ischemic stroke (AIS) and Transient Ischemic Attack (TIA). Our findings did not reveal a correlation between EASIX and stroke severity nor with the degree of disability or the outcomes at 12-month follow-up. Upon analyzing the correlation between EASIX and the subtypes of AIS, a significant association with disability severity and adverse outcomes in the lacunar infarcts (SVD) subgroup was observed, wherein elevated EASIX levels corresponded to greater disability at 12 months and heightened long-term mortality. Although based on a small sample (SVD = 10), this finding may point toward a potential link with the underlying pathophysiology of this subtype.

The stroke cohort was characterized by advanced age, with a median value of approximately 82 years. This finding reflects the age-related shift in the prevalence of stroke toward the later decades of life, as population aging is accompanied by the accumulation of risk factors such as hypertension, diabetes mellitus, and atrial fibrillation, as well as by progressive deterioration of vascular elasticity and endothelial function. According to large European studies, the median age at first-ever stroke typically ranges between 73 and 81 years. For instance, in the large multicenter population-based European Registers of Stroke (EROS) study, the median age at first stroke was reported to be around 73 years. In our cohort, the median age of 82 years is higher than most European reports and approaches that observed in the Dijon Stroke Registry (≈81 years). This difference may reflect demographic characteristics of the Mediterranean population as well as sampling particularities. Moreover, unlike most registries that include only first-ever strokes, our study included all stroke events, with approximately 37% of patients having a history of prior stroke. Consequently, the inclusion of recurrent events tends to increase the overall median age of our sample [25,26,27,28].

Our correlation analysis revealed that female patients demonstrated lower EASIX values, indicating potential sex-related variation in endothelial stress responses. Large-scale cohort investigations suggest that sex appears to influence the prognostic implications of EASIX. In the NHANES study, a significant interaction between sex and EASIX was observed (*p* interaction = 0.03), suggesting sex-specific differences in the relationship between EASIX and all-cause mortality. In the sepsis cohort derived from the MIMIC-IV database, EASIX showed an association with mortality in patients with sepsis. Higher EASIX levels were associated with an increased risk of death, an association that was more pronounced in males [29].

In our cohort, we also observed a positive association between WBC count, CRP, and EASIX; EASIX correlated positively with both WBC (B = 0.022, *p* = 0.001) and CRP (CRP: B = 0.023, *p* = 0.01). These findings could underscore the contribution of systemic inflammation to endothelial stress in cerebrovascular syndromes. In contrast to our findings, the study on critically ill patients with heart failure by Yin and Wang showed that the prognostic impact of elevated log_2_-EASIX on 1-year mortality was significantly stronger in patients with lower white blood cell counts (*p* for interaction < 0.05), suggesting a potential modifying effect of leukocyte count on the prognostic value of EASIX [30]. In the sepsis cohort, EASIX remained an independent predictor of both 28-day and 90-day mortality even after adjustment for WBC count, suggesting that its prognostic value was not confounded by leukocyte levels. By contrast, in our cohort we observed a positive correlation between WBC count and EASIX, indicating that systemic inflammation may directly contribute to higher EASIX values in acute stroke. This difference highlights that, while EASIX captures endothelial stress across both conditions, in stroke its association with outcomes may at least partly be mediated by inflammatory burden as reflected by leukocyte count. Inflammation may not only represent a consequence of ischemic injury but could also act as a precipitating factor for vascular occlusion. Elevated CRP and leukocyte count, which contribute to higher EASIX values, can induce endothelial activation, oxidative stress, and platelet aggregation—mechanisms known to precede cerebral infarction. Conversely, ischemic brain injury itself triggers a secondary inflammatory cascade characterized by cytokine release, leukocyte recruitment, and endothelial disruption. Therefore, EASIX may capture a composite signal reflecting both pre-existing endothelial stress and post-ischemic inflammatory responses [31,32,33].

Lacunar stroke is characterized by subcortical infarcts of deep perforating arteries usually as a result of hypohyalinosis and microatheroma formation that can lead to the compromise of the vessel integrity [34,35]. Lacunar strokes are considered a form of cerebral small vessel disease (SVD), which is increasingly viewed as a systemic endothelial dysfunction syndrome—affecting cerebral, renal, and retinal vessels alike [36]. In our study, the observed association between EASIX and negative outcome in the subgroup of lacunar strokes could suggest that EASIX may reflect an underlying endothelial dysfunction and microvascular injury. As shown recently by Knottnerus et al., endothelial dysfunction is also thought to play an important role in the pathophysiology of lacunar stroke, especially in patients with concomitant silent lacunar strokes and ischemic white matter lesions [37]. The role of endothelial dysfunction in this process is shown by the disruption of the regulation of vascular tone, promotion of inflammation, increase in permeability of the blood–brain barrier, and impairment of antithrombotic balance. These alterations contribute to the thickening of the vessel wall, reduced vasoreactivity, and ultimately luminal narrowing or occlusion. This process makes endothelial dysfunction not only a marker but a driver of the microvascular injury in the pathophysiology of lacunar stroke.

In the general AIS population, our findings failed to correlate EASIX levels with NIHSS or mRS at any timepoint, nor could they predict the 12-month mortality. These facts could suggest that EASIX may not be able to reflect stroke severity in acute phase, nor prognosis in the large artery atherosclerosis or cardioembolic strokes. But when taking into account the fact that EASIX was found to predict the outcome in hematologic patients who develop post-transplantation microangiopathy due to aGVHD where microvascular injury is present, our findings could support the hypothesis that EASIX may have greater prognostic value specifically in stroke subtypes characterized by microvascular pathology, such as small vessel disease (SVD), rather than those driven by macrovascular mechanisms. This observation likely reflects the unique pathophysiological background of SVD, which is characterized by chronic microvascular endothelial dysfunction and low-grade inflammation. In the setting of an acute ischemic event, this pre-existing endothelial impairment may become further exacerbated, leading to a more pronounced elevation of EASIX during the acute phase. Thus, the higher EASIX observed in SVD does not necessarily indicate more extensive ischemic injury but rather reflects the synergistic effect of chronic small-vessel pathology and acute endothelial stress associated with AIS.

Our findings do not align with the previous published research, where it was reported higher EASIX levels are correlated with increased stroke prevalence and all-cause mortality in all stroke subtypes. Apart from that, our work provides an additional contribution by specifically evaluating the association between EASIX and functional status in lacunar strokes, thereby bringing out the role of endothelial dysfunction in microangiopathic mechanisms.

The simplicity and accessibility of EASIX, which is easily calculated by the routine bloodwork during admission without the need for further specialized testing, adds further to its clinical relevance. This biomarker may offer a fast and relatively accurate tool for risk stratification of patients with lacunar stroke. Further, future research could even suggest EASIX as a diagnostic tool for early identification of lacunar strokes.

This was a pilot retrospective single-center study with a modest sample size (particularly SVD, *n* = 10), which limits the ability to generalize our findings. No multivariable adjustment was made due to limited power. Also, EASIX could be a rather volatile biomarker, affected by various parameters such as renal dysfunction, hematologic parameters, or infection, which may confound associations, leading to further bias. Given the limited number of SVD cases, these findings should be interpreted with caution and considered exploratory. Therefore, future prospective multicenter studies should be designed to add validation to the prognostic role of this biomarker.

## 5. Conclusions

Our study suggests that EASIX, a readily accessible biomarker of endothelial dysfunction, may hold prognostic relevance in the outcome and severity of lacunar strokes, indicating a possible role of endothelial dysfunction in small vessel ischemia. While EASIX did not predict outcomes in the overall stroke cohort, its association with disability and mortality in small vessel occlusion points toward potential subtype-specific utility. The potential utility of EASIX as a prognostic biomarker in this specific subgroup could highlight its relevance for improving risk assessment and guiding future research on individualized management approaches. Nonetheless, further large-scale, prospective, population-based studies are warranted to validate these results and clarify the role of EASIX in routine stroke evaluation.

## Figures and Tables

**Table 1:** Baseline characteristics. Numbers represent median values and interquartile range (Q1–Q3) for continuous variables, and absolute counts and proportions for categorical variables.

Variables	Acute Ischemic Stroke	Transient Ischemic Attack
	*n* = 71	*n* = 29
Age, years	82 (77–88)	82 (75–87)
Female sex	43 (60.6)	13 (44.8)
Arterial Hypertension	64 (90.1)	24 (82.8)
Diabetes Mellitus	28 (39.4)	7 (24.1)
Dyslipidemia	50 (70.4)	19 (65.5)
Atrial Fibrillation	26 (36.6)	8 (27.6)
Coronary Artery Disease	19 (26.8)	6 (20.7)
Peripheral artery disease	17 (23.9)	2 (6.9)
Previous Stroke	22 (31.1)	15 (51.7)
Current Smoking	14 (19.7)	6 (20.7)
LDH, U/L	239 (195–294)	233 (209–277)
Creatinine, mg/dL	0.99 (0.83–1.27)	0.89 (0.82–1.07)
Platelets, K/mL	212 (175–278)	203 (182–234)
EASIX	1.06 (0.77–1.6)	1.06 (0.93–1.39)
LN EASIX	0.06 (−0.26–0.47)	0.06 (−0.07–0.33)
WBC, K/mL	8.51 (6.61–10.43)	7.78 (6.69–9.42)
CRP, mg/dL	0.58 (0.19–2.05)	0.45 (0.11–1.82)
NIHSS at admission	8 (3–15)	2 (1–3)
mRS before admission	2 (1–3)	2 (1–3)
mRS at admission	5 (4–5)	3 (1–4)

**Table 2:** Correlation of EASIX with baseline characteristics.

Variables	B	*p* Value
Age	0.001	0.76
Sex (f)	−0.115	0.03
Arterial Hypertension	0.025	0.76
Diabetes Mellitus	−0.042	0.46
Dyslipidemia	0.062	0.28
Atrial Fibrillation	0.001	0.99
Peripheral Arterial Disease	−0.030	0.66
Coronary Artery Disease	0.095	0.12
Stroke History	0.046	0.41
Current Smoking	0.033	0.62
CRP	0.023	0.01
WBC	0.022	<0.001
LDL-cholesterol	0.001	0.85

**Table 3:** Correlation of EASIX with stroke severity and outcomes.

Outcomes	AIS (*n* = 71)		TIA (*n* = 29)	
	B	*p* Value	B	*p* Value
Days of Hospital Stay	0.001	0.94	0.003	0.89
NIHSS at Admission	−0.054	0.9	−0.256	0.73
NIHSS at Discharge	0.594	0.7	−0.661	0.32
mRS at Admission	0.001	0.99	0.314	0.48
mRS at Discharge	−0.026	0.93	0.531	0.26
In-Hospital Death	0.08	0.35	0.054	0.48
Death at 12 Months	0.061	0.54	0.116	0.3

B indicates regression coefficients derived from univariable regression analyses. Linear regression was used for continuous outcomes, and logistic regression was used for binary outcomes. Statistical significance was set at *p* < 0.05.

**Table 4:** Correlation of EASIX with outcomes on Non-SVD and SVD.

Outcomes	Non-SVD (*n* = 61)		SVD (*n* = 10)	
	B	*p* Value	B	*p* Value
Days of Hospital stay	0.2	0.89	0.741	0.9
NIHSS at Admission	−0.907	0.57	1.078	0.54
NIHSS at Discharge	−0.309	0.85	2.287	0.3
mRS at Admission	−0.066	0.75	0.175	0.78
mRS at 12 Month	−0.287	0.433	2.383	0.02
In-Hospital Death	0.055	0.55	0.261	0.33
Death at 12-month	−0.020	0.85	0.653	0.02

B indicates regression coefficients derived from univariable regression analyses. Linear regression was used for continuous outcomes, and logistic regression was used for binary outcomes. Statistical significance was set at *p* < 0.05.

## Data Availability

The data presented in this study are not available due to ethical reasons.
